# Active Detection of Glucose Metabolism Disorders Prior to Coronary Artery Bypass Grafting: Associations with In-Hospital Postoperative Complications

**DOI:** 10.3390/jcm14093123

**Published:** 2025-04-30

**Authors:** Alexey N. Sumin, Natalia A. Bezdenezhnykh, Ekaterina. V. Belik, Andrew V. Bezdenezhnykh, Olga V. Gruzdeva, Olga L. Barbarash

**Affiliations:** 1 Federal State Budgetary Scientific Institution “Research Institute for Complex Issues of Cardiovascular Diseases”, Academician L.S. Barbarash Boulevard, 6, Kemerovo 650002, Russia; n_bez@mail.ru (N.A.B.); sionina.ev@mail.ru (E.V.B.); o_gruzdeva@mail.ru (O.V.G.); olb61@mail.ru (O.L.B.); 2 Limited Liability Company “Family Health and Reproduction Center Krasnaya Gorka”, Suvorova St., 3A, Kemerovo 650044, Russia; andrew22014@mail.ru; 3 Federal State Budgetary Educational Institution of Higher Education, Kemerovo State Medical University, Voroshilova St., 22A, Kemerovo 650056, Russia

**Keywords:** coronary surgery, glucose intolerance, prediabetes, screening, postoperative complications, HbA1c

## Abstract

**Background/Objectives:** Patients with coronary artery disease undergoing coronary artery bypass grafting (CABG) have a high prevalence of type 2 diabetes mellitus (T2DM) and prediabetes. Glucose metabolism disorders (GMDs) are often asymptomatic and remain undetected, but untreated they can have adverse effects. To evaluate the possibilities of active screening in identifying T2DM and prediabetes before CABG and to assess the impact of GMD on the incidence of postoperative complications. **Methods**: This study included 1021 patients who underwent CABG in 2016–2018 at the department of cardiovascular surgery, whose glycemic status was determined. All patients had their glycated hemoglobin (HbA1c) levels measured; those without a previous diagnosis of diabetes underwent an oral glucose tolerance test. The frequency of newly diagnosed diabetes and prediabetes was evaluated. Postoperative complication rates were analyzed among patient groups with various types of GMDs and normal blood glucose levels. **Results:** Screening before CABG increased the number of patients with established type 2 diabetes from 20.9 to 27.8% and the number of people with prediabetes from 2.7% to 31.7%. When analyzing hospital complications, patients with type 2 diabetes compared to patients with normoglycemia were significantly more likely to develop heart failure (*p* = 0.010), multiple organ failure (*p* = 0.002), require extracorporeal homeostasis correction (*p* = 0.011), and wound dehiscence (*p* = 0.004). Nine patients (0.9%) died following CABG without being discharged from the hospital, with 90% of these deaths occurring in patients with GMDs. Any GMD (diabetes or prediabetes) was associated with an increased incidence of postoperative heart failure (OR 1.259; *p* = 0.011), rhythm disturbances (OR 1.236; *p* = 0.010), major cardiovascular complications and/or heart failure (OR 1.193; *p* = 0.039), and all cardiovascular complications (OR 1.455; *p* = 0.002). In the presence of any GMD, the risk of multiple organ failure increased by 2.5 times (OR 2.506; *p* = 0.014), extracorporeal correction of homeostasis increased by 1.8 times (OR 1.821; *p* = 0.034), and diastasis of the wound edges increased by 1.3 times (OR 1.266; *p* = 0.005). It is important that, when adjusting for gender and age, the effect of GMD on the described complications remained significant. **Conclusions**: Active preoperative detection established an extremely high prevalence of GMD in patients with multivessel coronary artery disease (59.5%). T2DM and prediabetes are significant predictors of postoperative complications of coronary artery bypass grafting.

## 1. Introduction

Diabetes mellitus is a well-known risk factor for cardiovascular disease and mortality [1]. The global prevalence of type 2 diabetes mellitus (T2DM) is estimated at 9.3% (463 million people) worldwide and will increase by 25% in 2030 and 51% in 2045 [2]. This makes T2DM an important clinical and prognostic factor when combined with cardio-vascular disease [3,4,5,6]. In addition to the prognostic impact of such a combination, it also has implications for patient treatment. For example, when choosing the tactics of myocardial revascularization, it is necessary to take into account the presence of diabetes in the patient: in such patients, open myocardial revascularization is preferable to PCI [7,8]. At the same time, the presence of diabetes affects both the immediate results of coronary artery bypass grafting (CABG) [9] and the long-term treatment outcomes [6]. Based on this fact, several questions arise that are currently being actively studied. First, what should be the tactics of perioperative patient management? There is conflicting evidence here: on the one hand, strict glucose control led to an increase in adverse events in the perioperative period [9,10]; on the other hand, a decrease in perioperative variability of glucose levels was associated with a decrease in the number of perioperative complications [10]. Based on these data, a second question arises—is it necessary to preoperatively detect not only diabetes mellitus but also other glucose metabolism disorders referred to as prediabetes? On the one hand, the detection of diabetes may allow for more adequate tactics of perioperative management of patients (including the choice of myocardial revascularization method); on the other hand, is there a need to detect prediabetes, can its detection and presence affect the outcomes of operations? Previous studies [11,12,13] did not provide an unambiguous answer to this question. Thus, in the work of Djupsjo C et al. [11], during long-term postoperative observation of patients, no effect of prediabetes or newly diagnosed diabetes before CABG on long-term survival was found compared to patients with normoglycemia. At the same time, another study showed that strict glucose control during CABG was associated with an increased risk of in-hospital mortality among patients with diabetes and with a reduced risk of serious complications among patients with newly diagnosed diabetes [9]. This served as the basis for the present study, the aim of which was to evaluate the feasibility of active diagnosis of glucose metabolism disorders before CABG surgery and their association with hospital perioperative complications.

## 2. Materials and Methods

### 2.1. Study Design

**Figure 1:**
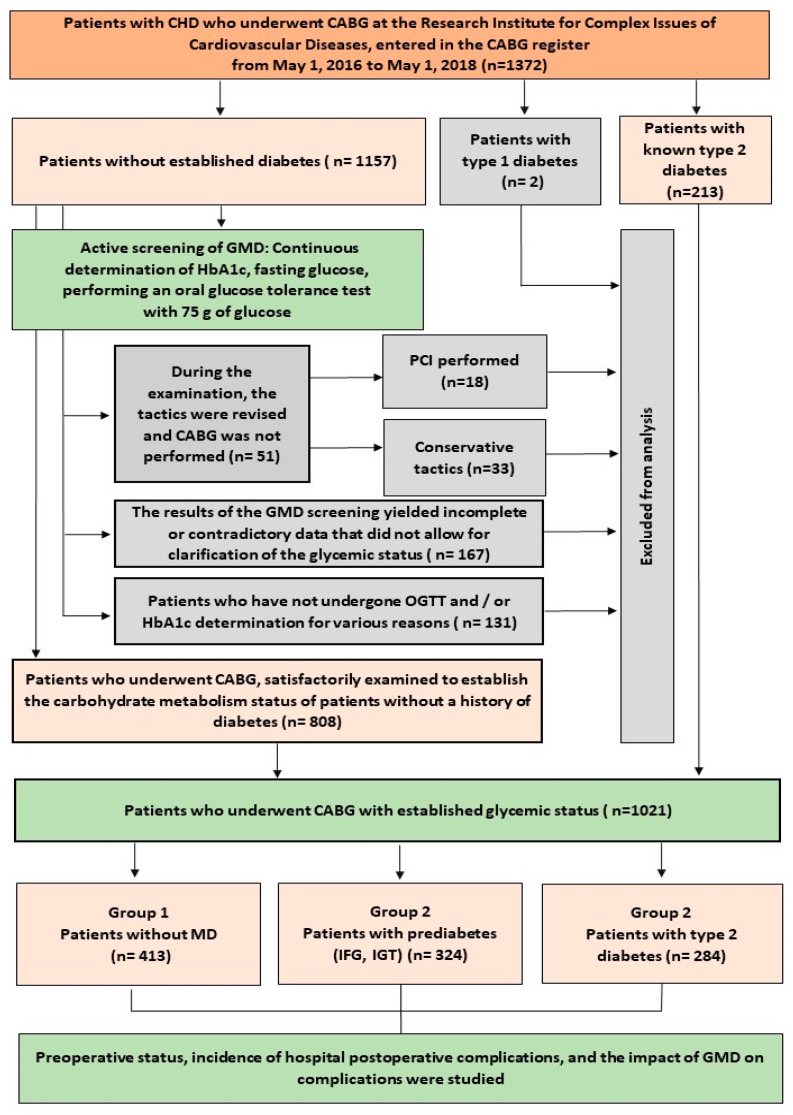
Study design. Notes: CHD—coronary heart disease; CABG—coronary artery bypass grafting; GMDs—glucose metabolism disorders; HbA1c—glycated hemoglobin, fraction C; OGTT—oral glucose tolerance test; PCI—percutaneous coronary intervention; IFG—impaired fasting glucose; IGT—impaired glucose tolerance.

The data of patients admitted to the Research Institute for Complex Issues of Cardiovascular Diseases for planned coronary artery bypass grafting from 1 May 2016 to 1 May 2018 (n = 1372) were analyzed. In addition to the traditional preoperative examination, patients without established diabetes mellitus were examined for latent GMD (diabetes mellitus and prediabetes; Figure 1). The study protocol was approved by the Local Ethical Committee of Research Institute for Complex Issues of Cardiovascular Diseases (Protocol No. 20160421; date of approval, 21 April 2016). Informed consent was obtained from all subjects involved in this study. All patients had their fasting blood glucose, glycated hemoglobin (HbA1c) determined, and most patients underwent an oral glucose tolerance test (OGTT) in the absence of contraindications. The patient’s attending physician entered the results obtained into the fields of the electronic program “Screening of glucose metabolism disorders before coronary artery bypass grafting” (hereinafter referred to as the “program”). This program is an algorithm for diagnosing glucose metabolism disorders, and, based on the totality of the data, it makes a diagnosis of “diabetes mellitus”, “impaired fasting glycaemia”, “impaired glucose tolerance” (or a combination thereof), or “normoglycemia”. The program was developed by the authors [14] and operates on the basis of the Coronary Artery Bypass Grafting Registry within the framework of the medical portal of the Research Institute for Complex Issues of Cardiovascular Diseases. The program has a wide variability in the ability to diagnose glucose metabolism disorders—it is possible to enter any previously known or current glucose results that will help establish a diagnosis even with ambiguous or insufficient current data. At the same time, the interpretation of the examination results is programmed in accordance with the current global and national recommendations for the diagnosis of diabetes mellitus and other glycemic disorders approved at that time [15,16]. Throughout the diagnostic process, the program provides recommendations on further actions before the operation; if necessary, the patient is referred to a specialist—an endocrinologist. The examination data within the GMD screening program are automatically entered into the Coronary Artery Bypass Grafting Registry. Since the authors took an active part in developing and maintaining the registry, this allowed the formation of a full-fledged database for research. If necessary, the registry was revised and additional work was carried out to enter parameters from primary documentation.

### 2.2. Study Population

The study design and patient selection are presented in Figure 1. A total of 1372 patients were hospitalized for planned coronary artery bypass grafting. Patients with previously diagnosed type 2 diabetes (n = 213) and two patients with type 1 diabetes were not screened at baseline (patients with type 1 diabetes were excluded from further analysis). Thus, 1157 patients who did not have diabetes mellitus at baseline underwent active diagnostics of glucose metabolism disorders (Figure 1). Those patients whose tactics were revised during the examination and where CABG was not performed were excluded from further analysis (n = 51). Those with insufficient data to clarify the state of glucose metabolism were also excluded: 167 patients with incomplete or contradictory data from screening, and 131 patients who did not undergo OGTT and/or HbA1c, and the available indicators were insufficient to establish a diagnosis. Thus, 808 patients without a history of diabetes were satisfactorily examined to establish the status of glucose metabolism. Together with 213 patients with known type 2 diabetes, they formed the study sample—1021 patients with known glycemic status (Figure 1). For further analysis, patients were divided into three groups by glycemic status: Group 1—patients without glucose metabolism disorders; Group 2—patients with prediabetes; and Group 3—patients with type 2 diabetes. The diagnostic criteria for GMD are detailed in the following section (Section 2.3).

### 2.3. Diagnosis of Glucose Metabolism Disorders

Diabetes mellitus was diagnosed when the following parameters were detected: fasting blood glucose concentration ≥ 7.0 mmol/L (126 mg/dL), glycemia level 120 min after the oral glucose tolerance test (OGTT) ≥ 11.1 mmol/L (200 mg/dL), as well as random detection of blood glucose ≥ 11.1 mmol/L in the presence of characteristic symptoms of hyperglycemia (polydipsia, polyuria, general weakness). In cases where there were no signs of acute metabolic decompensation, the diagnosis of diabetes mellitus was confirmed based on two blood glucose measurements corresponding to the diabetic range or a single determination of the glycated hemoglobin (HbA1c) level ≥ 6.5% in combination with a single blood glucose measurement [1,2]. To identify prediabetes (impaired fasting glycemia, impaired glucose tolerance), the World Health Organization (WHO) criteria of 1999–2013 and those used in our country were used, in contrast to the stricter standards proposed by the American Diabetes Association. According to WHO recommendations, impaired glucose tolerance (IGT) was diagnosed at a fasting plasma glucose level of less than 7.0 mmol/L (126 mg/dL) and a glucose level 2 h after OGTT within 7.8–11.1 mmol/L (140–200 mg/dL). Impaired fasting glucose (IFG) was defined as a fasting plasma glucose level of 6.1 to 6.9 mmol/L (110–125 mg/dL) and, when data were available, a 2 h post-OGTT glucose level of <7.8 mmol/L (<140 mg/dL) [15,16]. A glycated hemoglobin (HbA1c) level of up to 6.0% was considered normal, and values in the range of 6.0–6.4% corresponded to prediabetes. Prediabetes was defined as the presence of impaired fasting glucose (IFG), impaired glucose tolerance (IGT), or a combination of both. The terms type 2 diabetes and T2DM (type 2 diabetes mellitus) are used interchangeably throughout this paper.

### 2.4. Outcomes

This study analyzed preoperative patient status, incidence of postoperative in-hospital complications, and their predictors. Major adverse cardiovascular events (MACEs) were defined as any occurrence of in-hospital mortality, myocardial infarction, or stroke. Serious cardiovascular complications following CABG included the following: in-hospital mortality, myocardial infarction, heart failure requiring inotropic support, percutaneous coronary intervention for acute coronary syndrome occurring during postoperative hospitalization, arrhythmias, cerebral stroke, and emergency lower extremity arterial surgery due to acute ischemic decompensation. All the above-mentioned complications were classified as significant complications. In addition, significant complications included the following: multiple organ dysfunction syndrome, need for extracorporeal hemocorrection, gastrointestinal bleeding, transient ischemic attacks, respiratory failure, pneumonia, drainage of the pleural cavity in case of hydrothorax or pneumothorax, and complications of the postoperative wound. Wound-related complications included wound dehiscence, tissue necrosis, and purulent complications requiring surgical exploration of the wound with re-suture, as well as resternotomy for mediastinitis or bleeding.

### 2.5. Statistical Analyses

Statistical processing was performed using the standard STATISTICA 8.0 software package (Dell Software, Inc., Round Rock, TX, USA). The distribution of quantitative data was checked using the Shapiro–Wilk test. Since the distribution of all quantitative characteristics differed from normal, they were described using the median with the upper and lower quartiles (25th and 75th percentiles). The Kruskal–Wallis, Mann–Whitney, and χ^2^ (chi-square) tests were used to compare groups. With a small number of observations, Fisher’s exact test with Yates’s correction was used. The Bonferroni correction was used to solve the problem of multiple comparisons. Thus, taking into account the number of degrees of freedom, the critical significance level *p* when comparing three groups was taken to be 0.017, in other cases—0.05. Logistic regression analysis was used to assess the relationship between glucose metabolism disorders and hospital complications.

## 3. Results

### 3.1. Baseline Characteristics

**Figure 2:**
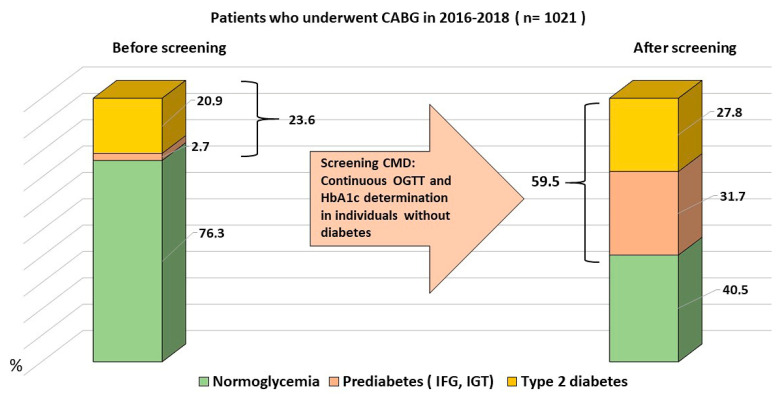
Prevalence of glucose metabolism disorders before and after targeted screening (n = 1021). Notes: T2DM—type 2 diabetes mellitus; GMDs—glucose metabolism disorders; CABG—coronary artery bypass grafting; HbA1c—glycated hemoglobin, fraction C; OGTT—oral glucose tolerance test; IFG—impaired fasting glucose; IGT—impaired glucose tolerance.

Before the GMD screening, 8 patients (0.8%) had isolated impaired fasting glycemia (IFG), 15 patients (1.5%) had isolated impaired glucose tolerance (IGT), 5 patients (0.5%) had a combination of IFG and IGT, and a total of 28 patients (2.7%) had known prediabetes before the screening. Type 2 diabetes mellitus (T2DM) diagnosed before the screening was diagnosed in 213 patients (20.9%) (Figure 2).

Following screening, a total of 284 cases of type 2 diabetes mellitus (T2DM) were identified (27.8%), including 72 newly diagnosed cases (7.1% of 1021 patients). Additionally, screening revealed 63 cases of isolated impaired fasting glycemia (IFG) (6.2%), 203 cases of isolated impaired glucose tolerance (IGT) (19.9%), and 58 cases of combined IFG and IGT (5.7%). The total number of prediabetes diagnoses was 324 (31.7%).

Preoperative screening significantly increased the detection of glucose metabolism disorders: diagnosed diabetes mellitus rose from 20.9% (n = 213) to 27.8% (n = 284); prediabetes cases increased from 2.7% (n = 28) to 31.7% (n = 324). Overall, the proportion of patients with any glucose metabolism disorder grew from 27.2% (n = 241) to 59.5% (n = 608). Consequently, only 40.5% (n = 413) of patients had normal glucose metabolism post-screening, compared to 76.3% prior to screening. Notably, 36.8% of all diabetes cases and 78.0% of prediabetes cases were identified exclusively through preoperative screening.

### 3.2. Preoperative Characteristics of Patients in Groups with or Without GMD

Table 1 presents the anamnestic and clinical characteristics of the patients. About 80% of patients in groups 1 and 2 were men. In group 3 (T2DM), there were significantly fewer men (63.7%) and significantly more women (36.3%) than in the other two groups (*p* < 0.001; Table 1). Patients with normoglycemia were significantly younger than patients with prediabetes and T2DM (*p* = 0.003 when comparing groups 1–2; *p* < 0.001 when comparing groups 1–3). Body mass index was the lowest in the normoglycemia group compared with the other two groups, and obesity and overweight were significantly less common in this group (*p* < 0.001 in all cases described; Table 1). The T2DM group had the lowest prevalence of smoking compared with the other two groups (*p* = 0.007 when comparing groups 1–3; *p* = 0.001 when comparing groups 2–3; Table 1). There were no differences in the severity of angina and heart failure, the prevalence of arterial hypertension (Table 1), unstable angina, or history of cardiovascular events (Supplementary Table S1). Perioperative risk assessed by EuroSCORE II was significantly higher in the T2DM group compared to the other two groups (*p* < 0.001 in both cases).

Hospital medical therapy before coronary artery bypass grafting is presented in Table 1 and Supplementary Table S2. Patients with normoglycemia were less likely to receive aspirin before CABG at the hospital stage compared to the prediabetes group (*p* < 0.001; Table 1). Patients with prediabetes were less likely to receive calcium channel blockers, loop diuretics, and mineralocorticoid receptor antagonists compared to the T2DM2 group (*p* = 0.018, *p* = 0.003, and *p* = 0.001, respectively; Table 1). In the T2DM group, 14.8% of patients received insulin prehospital and 48.6% in hospital before CABG (Supplementary Table S2).

When assessing preoperative laboratory parameters, HDL-C was significantly higher in the normoglycemic group compared to the T2DM group (*p* = 0.006), and triglycerides were significantly higher in both the T2DM and prediabetes groups compared to the normoglycemic group (*p* = 0.017 when comparing groups 1–2; *p* < 0.001 when comparing groups 1–3; Table 2). The median GFR according to CKD-EPI was significantly higher in the normoglycemic group compared to the T2DM group. Other routine parameters before CABG, except for glucose metabolism parameters, did not differ. The levels of glycated hemoglobin (HbA1c) and fasting venous blood glucose before CABG and on days 7–8 after CABG consistently increased from the group without CABG to the group with T2DM with statistical significance when comparing each of the three groups with each other (*p* < 0.001 in all cases; Table 2). The same trend was observed for blood glucose during the oral glucose tolerance test (*p* < 0.001 in all cases; Table 2).

According to the echocardiographic examination data (Table 3) before CABG, the left atrium size and left ventricular myocardial mass were significantly larger in the T2DM group compared to the normoglycemia group (*p* = 0.004 and *p* = 0.014, respectively). The data for the thickness of the interventricular septum and the posterior wall of the left ventricle were identical but did not reach significance (*p* = 0.022 and 0.068 for groups 1 and 3). The remaining echocardiographic parameters did not differ between the groups (Table 3).

When analyzing the coronary angiography data, the T2DM group had the least single-vessel disease compared to the other two groups (<0.001; Table 3). Moreover, patients with prediabetes had more three-vessel disease compared to the normoglycemic group (*p* = 0.002) and less two-vessel disease compared to the other two groups (<0.001 in both cases). Patients with left main coronary artery disease were more in the normoglycemic group compared to the T2DM group (*p* = 0.015). There were no differences in the number of significant stenosis of the brachiocephalic arteries and lower extremity arteries, or intima-media thickness (Supplementary Table S1).

The majority of CABG procedures were performed using cardiopulmonary bypass (CPB), with no significant intergroup differences observed (Table 4). Combined operations tended to be more frequent in groups with worse glucose metabolism: 22.8% (group 1), 27.8% (group 2), and 30.4% (group 3; *p* = 0.025 when comparing groups 1–3; insignificant with Bonferroni correction). The groups showed comparable rates of combined procedures, operative times, CPB durations, and cross-clamp times (Table 4).

### 3.3. Hospital Complications After Coronary Artery Bypass Grafting in Groups with or Without GMD

When analyzing hospital complications, patients with diabetes mellitus were significantly more likely to develop heart failure compared to patients with normoglycemia (*p* = 0.010; Table 5). For other cardiovascular complications, there was a statistically insignificant tendency for their number to increase in the prediabetes and diabetes groups compared to the normoglycemia group. In addition, patients with diabetes were significantly more likely to develop multiple organ failure syndrome (*p* = 0.002) and required extracorporeal hemostasis correction (*p* = 0.011); the differences were significant compared to the normoglycemia group.

Patients with T2DM were more likely to develop wound dehiscence (8.8%, 5.9, and 3.6% in groups 3, 2, and 1, respectively; *p* = 0.004 when comparing groups 1 and 3). For all other complications, there was a tendency for their frequency to increase from the normoglycemia group to the diabetes group but not for sternal wound bleeding and emergency lower limb surgery. This tendency did not reach statistical significance but was observed for almost all complications.

Patients with prediabetes and diabetes spent significantly longer in hospital after surgery than patients in the normoglycemia group (0.003 when comparing groups 1–3; *p* = 0.016 when comparing groups 1–2; Table 5). At the same time, the highest percentage of patients in the prediabetes group stayed in hospital after CABG for more than 10 days (*p* = 0.008 when comparing groups 1–2, 0.006 when comparing groups 2–3).

**Figure 3:**
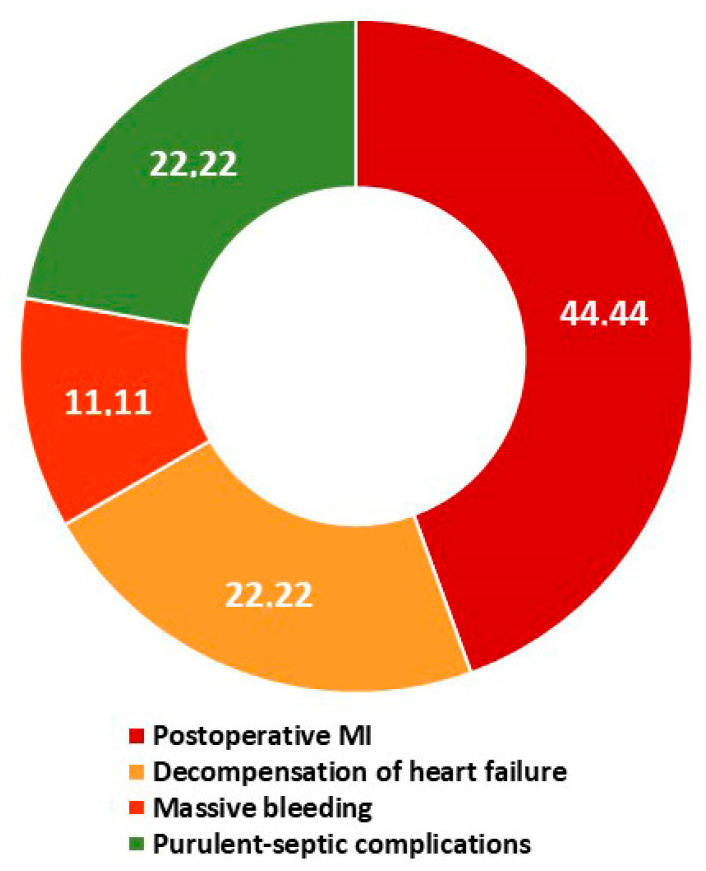
Causes of hospital deaths (n = 9). Notes: MI—myocardial infarction.

**Figure 4:**
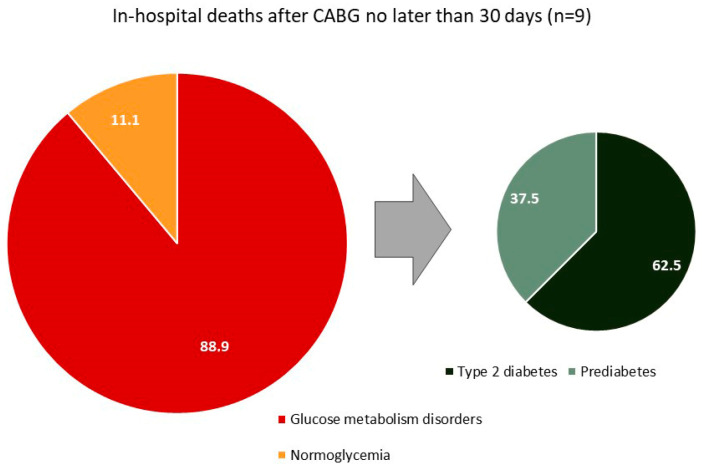
Structure of glucose metabolism disorders among those who died in hospital (n = 9).

The analysis of hospital mortality is presented in Table 5 and in Figure 3 and Figure 4. In-hospital mortality was understood as all cases of death from all causes in the postoperative period without hospital discharge but no later than 30 days after CABG. A total of nine patients died after CABG without hospital discharge, which amounted to 0.9% of the total number of patients (n = 1021). In the normoglycemia group, one patient died (0.2%), in the T2DM 2 group, five patients (1.8%), and, in the prediabetes group, three (0.9%) (Table 5). The causes of mortality are presented in Figure 3 (n = 9). Postoperative myocardial infarction caused four deaths (44.4%), decompensated chronic heart failure caused 22.2% of deaths (two cases), and purulent-septic complications in combination with multiple organ failure caused 22.2% (two cases). All of the deaths described above occurred in the diabetes or prediabetes group. The only death in the normoglycemia group was massive aortic bleeding with hemorrhagic shock (11.1% of all deaths). It should be noted that eight of the nine deceased patients had some type of glucose metabolism disorder (88.9%; Figure 3). Of these, 62.5% of patients had diabetes and 37.5% had prediabetes (Figure 4).

### 3.4. The Impact of Glucose Metabolism Disorders on the Development of Hospital Complications After CABG: Results of Logistic Regression Analysis

**Figure 5:**
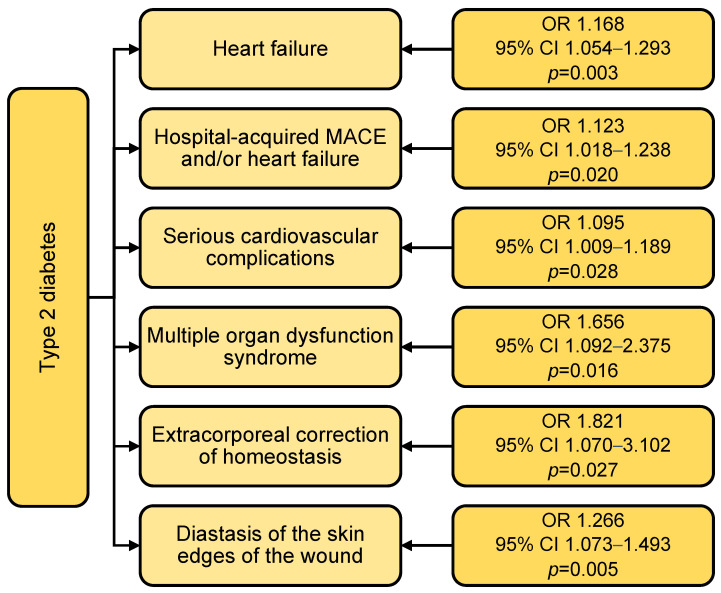
Impact of type 2 diabetes mellitus on the incidence of hospital complications of coronary artery bypass grafting. Notes: MACEs—major cardiovascular events (myocardial infarction and/or stroke and/or death in hospital); OR—odds ratio; CI—confidence interval.

According to the logistic regression data, type 2 diabetes mellitus increased the incidence of heart failure (OR 1.168; *p* = 0.003), major cardiovascular events and/or heart failure (OR 1.123; *p* = 0.020), serious cardiovascular complications (OR 1.095; *p* = 0.028) (Figure 5). In the presence of type 2 diabetes, the incidence of multiple organ failure increased by 66% (OR 1.656; *p* = 0.016), the incidence of extracorporeal hemostasis correction increased by 82% (OR 1.821; *p* = 0.027), and diastasis of the skin edges of the wound by 27% (OR 1.266; *p* = 0.005). Moreover, when adding gender and age, the effect of type 2 diabetes on all the described complications remained.

**Figure 6:**
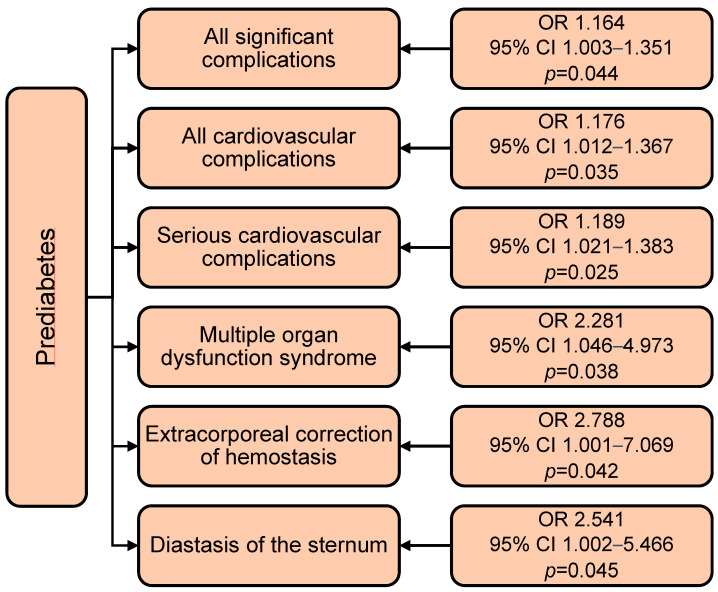
Impact of prediabetes on the incidence of in-hospital complications of coronary artery bypass grafting. Notes: OR—odds ratio; CI—confidence interval.

Prediabetes increased the incidence of all significant complications by 16% (OR 1.164; *p* = 0.044), all cardiovascular complications by 18% (OR 1.176; *p* = 0.035), and serious cardiovascular complications by 19% (OR 1.189) (Figure 6). The presence of prediabetes before CABG increased the incidence of multiple organ failure syndrome by 2.3 times (OR 2.281; *p* = 0.038), extracorporeal hemostasis correction by 2.8 times (OR 2.788; *p* = 0.042), and the risk of sternal diastasis by 2.5 times (OR 2.541; *p* = 0.045). It should be noted that, when adding gender and age, the effect of prediabetes decreased and acquired borderline significance, and age acquired the main predictive significance in the models for all the complications described.

**Figure 7:**
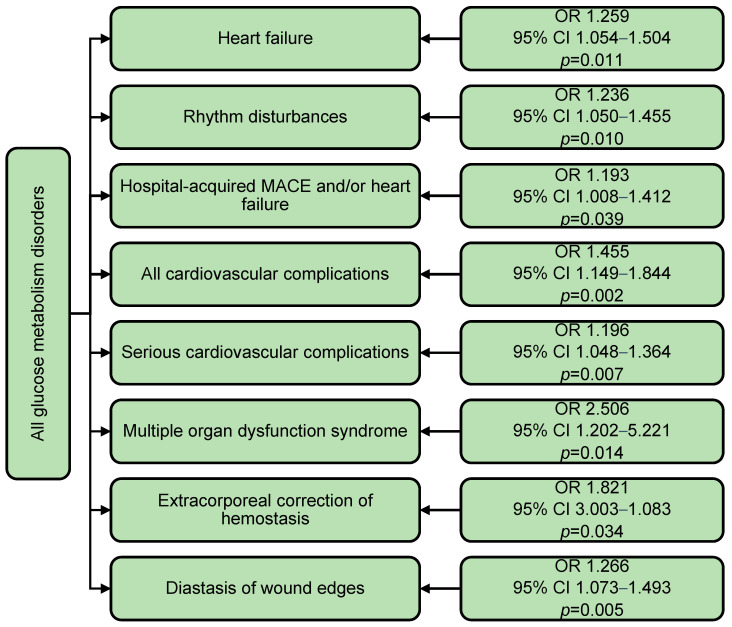
The impact of all glucose metabolism disorders on the incidence of in-hospital complications of coronary artery bypass grafting. Notes: GMDs—glucose metabolism disorders; MACEs—major cardiovascular events (myocardial infarction and/or stroke and/or death in hospital); OR—odds ratio; CI—confidence interval.

When analyzing glucose metabolism disorders as a binary variable (presence of type 2 diabetes or prediabetes), the spectrum of associated hospital complications broadened significantly (Figure 7). GMD was associated with increased risks of heart failure (OR 1.259; *p* = 0.011), arrhythmias (OR 1.236; *p* = 0.010), major cardiovascular events and/or heart failure (OR 1.193; *p* = 0.039), and all cardiovascular complications (OR 1.455; *p* = 0.002). Additionally, GMD elevated the risk of multiple organ failure by 2.5-fold (OR 2.506; *p* = 0.014), extracorporeal hemostasis correction by 82% (OR 1.821; *p* = 0.034), and wound edge separation by 27% (OR 1.266; *p* = 0.005) (Figure 7). After adjustment for sex and age, the association between GMD and complications remained significant for all outcomes except wound edge separation, where female sex became the primary predictor.

It is also important to highlight that pre-existing carbohydrate metabolism disorders (known prior to screening) showed no significant association with complications in the regression analysis. Furthermore, without screening, all newly diagnosed cases of diabetes and prediabetes would have remained undetected and, when assessing complications, would have been misclassified as part of the normoglycemia group.

## 4. Discussion

This study revealed a remarkably high prevalence of glucose metabolism disorders (GMDs) in a cohort of patients undergoing surgical treatment for coronary artery disease. Active preoperative screening prior to CABG increased the proportion of diagnosed diabetes mellitus cases from 20.9% to 27.8%, prediabetes from 2.7% to 31.7%, and overall GMD diagnoses from 27.2% to 59.5%. The presence of GMD was significantly associated with a higher incidence of postoperative complications, including multiple organ failure, cardiovascular events, and wound-related complications.

Screening for the detection of diabetes and prediabetes has been used in recent years in different cohorts of subjects [17,18]. For example, in the epidemiological study HUNT Study in Norway, when determining HbA1c in individuals over 20 years old, the prevalence of diabetes was 6.0%; 11.1% of cases of which were previously undiagnosed. At the same time, the prevalence of prediabetes was 6.4% [19]. Most often, screening for GMD is carried out during hospitalization of patients for various indications and is limited to determining the HbA1c level. Thus, when determining HbA1c in Croatian hospitals, the prevalence of prediabetes ranged from 14.2% to 20.5%, and undiagnosed diabetes from 3.3% to 7.3% [20]. During preoperative assessment, uncontrolled HbA1c levels (>7%) were detected in 54.7% of cases [21]. However, the work does not provide data on the frequency of newly diagnosed diabetes mellitus among the examined patients, and a small number of patients were included [21]. In patients with abdominal aortic aneurysm, HbA1c values ≥ 6.5% allowed us to identify an additional 25% of patients with diabetes in the ABANDIA study [22]. When screening HbA1c in patients over 65 years of age before non-cardiac surgeries, among patients without a diagnosis of diabetes, the prevalence of undiagnosed diabetes and prediabetes was 3.7% (95% CI 2.5–5.4%) and 42.9% (95% CI 39.2–46.7%), respectively [23]. The data of the present study are quite consistent with these results; some differences in the figures are due to a different cohort of patients (before CABG in our study) and the additional glucose tolerance test in our study.

The negative impact of diabetes mellitus on the immediate results of CABG has been shown in previous studies. However, the impact of the presence of borderline glucose metabolism disorders on the incidence of perioperative complications after CABG is not so clear. Therefore, the clinical significance of screening for glucose metabolism disorders in the preoperative period remains unclear. On the one hand, the detection of hidden glucose metabolism disorders in epidemiological studies makes it possible to identify a group of patients with an increased risk of developing cardiovascular pathology and a worse prognosis [24]. In addition, in the presence of cardiovascular diseases, the detection of diabetes mellitus and prediabetes during screening is associated with a subsequent unfavorable prognosis [13]. Therefore, such screening in patients in the preoperative period seems quite reasonable. However, there is still a significant lack of information assessing the impact of glycated HbA1c levels and its impact on mortality and morbidity after cardiac surgery [25].

However, the impact of newly identified glucose metabolism disorders on the immediate results of surgical treatment turned out to be contradictory. While a number of publications did not reveal an increase in the number of perioperative complications in such patients, such an association was still observed in others. Thus, the detection of diabetes and prediabetes during HbA1c screening in the preoperative period was not associated with an increased risk of 30-day mortality in non-cardiac surgeries [23]. Similar results were obtained in a recent study by Alshair F et al. [21]: a lack of effect of increased HbA1c on the immediate results of CABG surgery. This may be due to the small number of patients included in the study.

On the other hand, preoperative HbA1c levels above 7.5% were associated with poor perioperative glycemic control and more frequent episodes of dysglycemia. Higher preoperative HbA1c was found to be associated with increased postoperative hyperglycemia, acute kidney injury, intensive care unit admission, and longer hospital stay. The incidence of postoperative wound infection was also higher [26]. Significantly elevated preoperative HbA1c (≥8.5%) was associated with an increased risk of severe renal injury after CABG surgery [27]. Moreover, in patients without diabetes, HbA1c ≥ 6% was a significant independent predictor of early postoperative renal failure [28]. For such patients, delaying surgery to achieve optimal glycemic control seems reasonable, which further justifies the need to assess HbA1c before surgery. Interestingly, even the effect of glycemic control in the perioperative period may vary: with strict glycemic control in previously diagnosed diabetes, the risk of in-hospital mortality increases, while, in newly diagnosed diabetes, on the contrary, it reduces the risk of major complications [9]. The present study is consistent with the idea that detection of diabetes mellitus and prediabetes before CABG surgery allows us to identify a group of patients with GMD who have an increased risk of developing postoperative complications.

A previous meta-analysis showed that higher preoperative HbA1c levels could potentially increase the risk of surgical site infections, renal failure, and myocardial infarction in patients with diabetes after CABG and increase the risk of death and renal failure in patients without diabetes [29]. However, the authors of the meta-analysis note that there remain many inconsistencies in the definition of high HbA1c thresholds, and there is still a need for high-quality randomized clinical trials [29]. So far, only retrospective studies have been published in this area. Thus, the recent Diabetes and Infectious Outcomes in Cardiac Surgery (DOCS) study examined a new model of patient management in cardiac surgery. It consisted of preoperative HbA1c screening and glucose monitoring in patients with HbA1c ≥ 6% or with known diabetes. Daily management of glycemic therapy until discharge resulted in a reduction in perioperative infectious complications, including those from the surgical wound, in the HbA1c screening group [30]. Based on the results of the study, it seems reasonable to conduct a prospective randomized study to study the need for preoperative screening for GMDs.

Remote results of preoperative screening for glucose metabolism disorders are even more difficult to assess. Thus, the DACAB study assessed the effect of baseline HbA1c levels on vein graft patency after coronary artery bypass grafting (CABG). In the subgroup with a preoperative HbA1c level < 6.5%, higher vein graft patency was noted 1 year after CABG [31]. At the same time, survival and event-free survival were similar in patients with prediabetes and normoglycemia before CABG during the year after CABG [32]. In addition, at 10-year follow-up after surgery, survival in the groups with normoglycemia, prediabetes, and newly diagnosed diabetes was comparable, including after multivariate adjustment [11]. It is quite possible that this is because a timely diagnosis was accompanied by adequate treatment of diabetes in the postoperative period, which contributed to an improved prognosis in the long-term period. There may be numerous reasons for such results during long-term postoperative follow-up; one of them is shown in the study by Funamizu T, et al., where strict diabetes control, achieving an HbA1c level of <6.5 in a group of patients with diabetes after PCI, led to a worsening prognosis during 10-year follow-up [33].

The findings of this study support the implementation of routine preoperative screening for glucose metabolism disorders in all CABG candidates. The identification of previously undiagnosed diabetes or prediabetes could significantly influence clinical decision-making, particularly in borderline cases where revascularization strategy (CABG vs. PCI) remains debated. For instance, in patients with newly detected diabetes and multivessel disease, CABG may be preferred over PCI given its proven long-term survival benefits in this population. Such screening could be incorporated into preoperative pathways to optimize both glycemic control prior to surgery and long-term postoperative management.

### Study Limitations

This study was conducted in a single cardiac surgery unit, which may limit the extrapolation of the results to other patient populations and institutions with different treatment protocols. Second, in this study, we only assessed in-hospital outcomes after coronary artery bypass grafting without follow-up. This study did not evaluate whether active detection of GMD changed patient management (e.g., initiation of hypoglycemic therapy) and how this may have changed the incidence of complications. However, glycemic control was certainly performed in all patients if values exceeded target values. The definition of postoperative complications (e.g., “diastasis of wound margins”) may vary depending on clinical practice, potentially affecting the reproducibility of the results.

## 5. Conclusions

Screening of glucose metabolism disorders before CABG surgery additionally revealed newly diagnosed diabetes in 6.9% of patients and prediabetes in 29% of patients. Both the presence of diabetes mellitus and the presence of prediabetes were associated with an increase in the number of perioperative complications (primarily cardiovascular and complications from the surgical wound). The effect of active screening on the tactics of perioperative management of patients with GMD and the long-term prognosis requires further study.

## Figures and Tables

**Table 1:** Comparative preoperative characteristics by presence of glucose metabolism disorders (n = 1021).

Indicator	Group 1 Without GMD n = 413	Group 2 Prediabetes n = 324	Group 3 Type 2 Diabetes n = 284	*p*
Men (n, %)	340 (82.3)	264 (81.5)	181 (63.7)	<0.001 <0.001 _1–3, 2–3_
Women (n, %)	73 (17.68)	60 (18.5)	103 (36.3)	<0.001 <0.001 _1–3, 2–3_
Age (years, Me [LQ; UQ])	61.9 [56.6;66.9]	64.1 [58.8;68.1]	64.5 [59.9;68.8]	<0.001 0.003 _1–2_ <0.001 _1–3_
BMI (kg/m^2^, Me [LQ;UQ])	26.8 [24.2; 29.1]	27.7 [25.0; 30.7]	28,8 [26.0; 31.6]	<0.001 <0.001 _1–2, 1–3_
Obesity (BMI ≥30 kg/m^2^, n, %)	82 (19.9)	98 (30.2)	104 (36.6)	<0.001 <0.001 _1–2, 1–3_
Obesity or overweight (BMI ≥ 25 kg/m^2^, n, %)	263 (63.7)	244 (75.3)	232 (81.7)	<0.001 <0.001 _1–2, 1–3_
Arterial hypertension (n, %)	222 (53.8)	190 (58.6)	179 (63.0)	0.048
III—IV FC angina (n, %)	20 (4.8)	22 (6.8)	19 (6.7)	0.671
III FC CHF according to NYHA (n, %)	20 (4.8)	22 (6.8)	19 (6.7)	0.648
EuroSCORE II (%, Me LQ;UQ])	1.30 [0.85; 1.85]	1.31 [0.87; 1.88]	1.85 [1.06; 2.59]	<0.001 <0.001 _1–3, 2–3_
Hospital medical therapy in groups (n, %)				
Acetylsalicylic acid	368 (89.1)	322 (99.4)	251 (88.4)	<0.001 <0.001 _1–2_
Angiotensin-converting enzyme inhibitors	286 (69.2)	229 (70.7)	215 (75.7)	0.075
Angiotensin 2 receptor antagonists	126 (30.8)	94 (29.0)	68 (23.9)	0.087
β-blockers	362 (87.7)	286 (88.3)	257 (90.5)	0.134
Calcium channel blockers	262 (63.4)	189 (58.3)	192 (67.6)	0.018
Loop diuretics	181 (43.8)	127 (39.2)	145 (51.1)	0.005 0.003 _2–3_
Thiazide-like diuretics	182 (44.1)	132 (40.7)	141 (49.6)	0.146
Statins	378 (91.5)	276 (85.2)	254 (89.4)	0.172
Mineralocorticoid receptor antagonists	191 (46.2)	133 (41.0)	155 (54.6)	<0.001 0.001 _2–3_

Notes: GMD—glucose metabolism disorders; BMI—body mass index; FC—functional class; CHF—chronic heart failure; NYHA—New York Heart Association; Me [LQ; UQ]—median with upper and lower quartile; *p*—results of Kruskal–Wallis test; *p* _1–2, 2–3, 1–3_—*p* in pairwise comparison of groups 1–2, 2–3, 1–3.

**Table 2:** Baseline laboratory parameters according to glucose metabolism status (n = 1021), Me [LQ; UQ].

	Group 1 Without GMD n = 413	Group 2 Prediabetes n = 324	Group 3 Type 2 Diabetes n = 284	*p*
Total cholesterol (mmol/L)	4.5 [3.7; 5.4]	4.6 [3.8; 5.4]	4.45 [3.6; 5.9]	0.740
HDL cholesterol (mmol/L)	1.1 [0.92; 1.34]	1.05 [0.89;1.29]	1.02 [0.87;1.2]	0.006 _1–3_
LDL cholesterol (mmol/L)	2.67 [2.12; 3.41]	2.69 [2.15;3.53]	2.66 [1.90;3.76]	0.748
Triglycerides (mmol/L)	1.4 [1.07; 1.95]	1.55 [1.25;2.11]	1.76 [1.33;2.30]	<0.001 0.017 _1–2_ < 0.001 _1–3_
Creatinine (µmol/L)	76.0 [64.0; 88.0]	78.0 [68.0;97.0]	80.0 [65.0;92.0]	0.296
GFR according to CKD-EPI (ml/min/1.73 m^2^)	94.7 [82.2; 103.7]	92.9 [74.1; 101.1]	91.3 [76.0;98.4]	0.010 0.008 _1–3_
Glucose metabolism indicators				
HbA1c before CABG, %	5.2 [4.9;5.5]	5.8 [5.3;6.1]	7 [6.3;7.9]	<0.001 <0.001 _1–2, 2–3, 1–3_
Fasting venous blood glucose before CABG, mmol/L	5.1 [4.8; 5.5]	5.8 [5.2; 6.2]	7.3 [6.0; 8.8]	<0.001 <0.001 _1–2, 2–3, 1–3_
Fasting glucose according to OGTT, mmol/L	4.6 [4.3;5.2]	5.2 [4.6;5.7]	6.2 [5.5;6.8]	<0.001 <0.001 _1–2, 2–3, 1–3_
Glucose 2 h after load according to the OGTT data, mmol/L	5.8 [4.9;6.7]	7.6 [5.9;8.7]	11.2 [8.6;11.9]	<0.001 <0.001 _1–2, 2–3, 1–3_

Notes: All data are presented as Me [LQ; UQ]— median with upper and lower quartiles; GMD—glucose metabolism disorders; HDL—high density lipoproteins; LDL—low density lipoproteins; GFR—glomerular filtration rate; CKD-EPI—Chronic Kidney Disease Epidemiology Collaboration; OGTT—oral glucose tolerance test; *p*—results of Kruskal–Wallis test; *p* _1–2, 2–3, 1–3_—*p* in pairwise comparison of groups 1–2, 2–3, 1–3.

**Table 3:** Data of instrumental examinations in groups with or without GMD.

	Group 1 Without GMD n = 413	Group 2 Prediabetes n = 324	Group 3 Type 2 Diabetes n = 284	*p*
Echocardiography, Me [LQ; UQ])				
LV end-diastolic volume, mL	154.0 [135.0;187.0]	154.0 [135.0;194.0]	154.0 [92.0;374.0]	0.640
LV end-diastolic dimension, cm	5.6 [5.3; 6.1	5.6 [5.3; 6.2]	5.6 [4.5; 8.3]	0.660
LV end-systolic volume, mL	61.0 [49.0;93.0]	62.0 [52.0;91.0]	67.0 [53.0;104.0]	0.291
LV end-systolic dimension, cm	3.7 [3.4;4.3]	3.7 [3.4;4.4]	3.7 [2.8;7.4]	0.541
Left atrium, cm	4.3 [3.9; 4.6]	4.3 [4.0; 4.7]	4.4 [3.5; 6.4]	0.014 0.004 _1–3_
Interventricular septum, mm	1.1 [1,0;1.2]	1.1 [1.0;1.2]	1.1 [0.7;1.6]	0.022
LV posterior wall, mm	1.0 [1.0;1.2]	1.1 [1.0;1.2]	1.1 [0.7;1.6]	0.068
Right ventricle, mm	1.9 [1.8;2.0]	1.9 [1.8;2.0]	1.0 [1.0;3.4]	0.135
Aorta, cm	3.6 [3.4;3.8]	3.6 [3.3;3.8]	3.5 [3.3;3.7]	0.348
LV ejection fraction, %	62.0 [52.0;66.0]	62.0 [54.0;65.0]	62 [51.0;66.0]	0.623
LV myocardial mass according to Deveraux and Reichek, g	293.8 [150.5;187.0]	310.7 [258.3;376.0]	316.5 [267.2;390.2]	0.016 0.014 _1–3_
Coronary angiography results (n, %)				
1 vessel disease *	82 (19.9)	64 (19.8)	21 (7.4)	<0.001 <0.001 _1–3, 2–3_
2 vessels disease *	175 (42.4)	85 (26.2)	122 (42.9)	<0.001 <0.001 _1–2, 2–3_
3 vessels disease *	169 (40.9)	170 (52.5)	132 (46.5)	0.002 0.003 _1–2_
Stenosis of the left main coronary artery > 50%	95 (23.0)	66 (20.4)	44 (15.5)	0.015 <0.001 _1–3_

Notes: GMD—glucose metabolism disorders; LV—left ventricle; *—number of affected main coronary arteries; Me [LQ; UQ]—median with upper and lower quartiles; *p*—results of Kruskal–Wallis test; *p* _1–2, 2–3, 1–3_—*p* in pairwise comparison of groups 1–2, 2–3, 1–3.

**Table 4:** Operative characteristics and preoperative risk assessment of CABG stratified by glucose metabolism status.

	Group 1 Without GMD n = 413	Group 2 Prediabetes n = 324	Group 3 Type 2 Diabetes n = 284	*p*
Cardiopulmonary bypass (n, %)	363 (87.9)	294 (90.7)	257 (90.4)	0.259
Off pump (n, %)	49 (11, 9)	28 (8.6)	26 (9.2)	0.296
Off-pump to on-pump conversion (n, %)	1 (0.2)	2 (0.6)	1 (0.3)	0.715
Isolated CABG (n, %)	319 (77.2)	234 (72.2)	198 (69.7)	0.025 _1–3_
Combined operations (n, %)	94 (22.8)	90 (27.8)	86 (30.4)	0.025 _1–3_
Carotid endarterectomy (n, %)	2 (0.5)	2 (0.6)	3 (1.1)	0.938
Ventriculoplasty (n, %)	21 (5.1)	13 (4.0)	6 (2.1)	0.097
Radiofrequency ablation (n, %)	2 (0.5)	0 (0)	3 (1.1)	0.227
Mitral valve (n, %)	2 (0.5)	3 (0.9)	4 (1.4)	0.987
Aortic valve (n, %)	0 (0)	2 (0.6)	2 (0.7)	0.218
Combined hybrid intervention (n, %)	1 (0.2)	0 (0)	1 (0.4)	0.654
CPB duration (minutes, Me [LQ; UQ])	84.5 [72.0;105.0]	81 [67.0;104.0]	85.5 [68.0;109.0]	0.489
Aortic clamping time (minutes, Me [LQ;UQ])	55.0 [45.0;71.0]	54 [44.0; 70.0]	54.0 [43.0; 73.0]	0.857
Cardioplegia frequency, Me [LQ;UQ])	2 [2; 3]	2 [2; 3]	2 [2; 3]	0.340
Total duration of the operation (hours, Me [LQ;UQ])	3.4 [3.1; 4.2]	3.5 [3.1; 4.3]	3.5 [3.2; 4.3]	0.302
Number of shunts (Me [LQ;UQ])	2 [2; 3]	2 [2; 3]	3 [2; 3]	0.217
Number of distal anastomoses (Me [LQ;UQ])	2 [2; 3]	2 [2; 3]	3 [2; 3]	0.364

Notes: GMDs—glucose metabolism disorders; Me [LQ; UQ]—median with upper and lower quartiles; CPB—cardiopulmonary bypass; *p*—results of Kruskal–Wallis test; *p* _1–2, 2–3, 1–3_—*p* in pairwise comparison of groups 1–2, 2–3, 1–3.

**Table 5:** Comparison of postoperative complications by glucose metabolism status.

	Group 1 Without GMD n = 413	Group 2 Prediabetes n = 324	Group 3 Type 2 Diabetes n = 284	*p*
All significant complications	206 (49.9)	187 (57.7)	148 (52.1)	0.111
Hospital MACE	10 (2.4)	11 (3.4)	12 (4.2)	0.399
Hospital MACE + HF	62 (15.0)	60 (18.5)	61 (21.5)	0.078
Serious cardiovascular complications	111 (26.9)	107 (33.0)	98 (34.6)	0.059
All cardiovascular complications	137 (33.2)	133 (41.1)	114 (40.1)	0.053
Emergency PCI for ACS	0 (0)	2 (0.1)	3 (0.1)	0.132
Arrhythmias	65 (15.7)	75 (23.2)	60 (21.1)	0.031
Heart failure	52 (12.6)	53 (16.4)	60 (21.1)	0.010 0.003 _1–3_
Myocardial infarction	3 (0.7)	4 (1,2)	6 (2.1)	0.275
Stroke	5 (1,2)	5 (1.5)	4 (1.4)	0.991
Transient ischemic attack	2 (0.5)	1 (0.3)	2 (0.7)	0.782
Emergency surgery on the arteries of the lower extremities	4 (1.0)	3 (0.9)	1 (0.4)	0.419
Multiple organ failure syndrome	2 (0.5)	8 (2.5)	10 (3.5)	0.013 0.002 _1–3_
Extracorporeal hemocorrection	1 (0.2)	6 (1.8)	7 (2.5)	0.016 0.011 _1–3_
Gastrointestinal bleeding	1 (0.2)	1 (0.3)	2 (1,1)	0.269
Intestinal obstruction	0 (0)	2 (0.6)	1 (0.3)	0.300
Progression of renal failure in CKD	9 (2.2)	9 (2.8)	11 (3.9)	0.413
Diastasis of skin wound edges	15 (3.6)	19 (5.9)	25 (8.8)	0.015 0.004 ^1−3^
Diastasis of the sternum	1 (0.2)	5 (1.5)	5 (1.8)	0.099
Remediastinotomy for mediastinitis	2 (0.5)	3 (0.9)	2 (0.7)	0.770
Remediastinotomy for bleeding	9 (2.2)	4 (1,2)	7 (2.5)	0.502
Pneumonia	31 (7.5)	27 (8.4)	26 (9.2)	0.727
Respiratory failure	9 (2.2)	15 (4.4)	14 (5.0)	0.097
Death in hospital	1 (0.2)	3 (0.9)	5 (1.8)	0.106
Hospital stays after CABG, days (Me [LQ; UQ])	13.0 [11.0; 17.0]	14.0 [12.0; 21.0]	14.0 [12.0; 20.0]	0.006 0.003 _1–3_ 0.016 _1–2_
Hospital stay after CABG > 10 days (n, %)	317 (76, 7)	274 (84.6)	215 (75.7)	0.013 0.008 _1–2_ 0.006 _2–3_

Notes: GMDs—glucose metabolism disorders; MACEs—major cardiovascular events (myocardial infarction and/or stroke and/or death in hospital); HF—heart failure; ACS—acute coronary syndrome; PCI—percutaneous coronary intervention; CKD—chronic kidney disease; *p*—results of Kruskal–Wallis test; *p* _1–2, 2–3, 1–3_—*p* in pairwise comparison of groups 1–2, 2–3, 1–3.

## Data Availability

The original contributions presented in this study are included in the article. Further inquiries can be directed to the corresponding author.

## Supplementary Materials

The following supporting information can be downloaded at: https://www.mdpi.com/article/10.3390/jcm14093123/s1. Table S1: Additional data from anamnesis and instrumental examination; Table S2: Medical therapy for diabetes mellitus (prehospital *).
