# Supplementary material for: Active Detection of Glucose Metabolism Disorders Prior to Coronary Artery Bypass Grafting: Associations with In-Hospital Postoperative Complications
Source: J Clin Med. 2025 Apr 30;14(9):3123. doi: 10.3390/jcm14093123 (PMC12072462; doi:10.3390/jcm14093123)
Supplement: Supplementary file 1 [file jcm-14-03123-s001.zip › jcm-3569570-supplementary.pdf]

Supplementary Table S1. Additional data from anamnesis and instrumental examination

Index (n, %) *	Group 1 Without CMD n =413	Group 2 Prediabetes n = 324	Group 3 Type 2 diabetes n =284	p
Rhythm disturbances	132 (32.0)	85 (26.2)	94 (33.1)	0.216
Implantation of pacemaker	0 (0)	2 (0.6)	3 (1.1)	0.133
Unstable angina	5 (1.2)	4 (1.2)	6 (2.1)	0.565
Intermittent claudication	46 (11.1)	38 (11.7)	37 (13.0)	0.633
Smoking status				
Smoking at the time of surgery	67 (16.2)	59 (18.2)	26 (9.1)	0.007 [1-3]<br>0.001 [2-3]
Never smoked	297 (71.9)	219 (67.5)	221 (77.8)	0.005 [1-2]
Threw it away	49 (11.9)	46 (14.1)	37 (13.0)	0.408
History of cardiovascular events and interventions				
Myocardial infarction in anamnesis	166 (39.7)	129 (39.8)	119 (41.9)	0.652
Stroke in anamnesis	19 (4.6)	10 (3.1)	16 (5.6)	0.302
Percutaneous coronary intervention	46 (11.1)	36 (11.1)	31 (10.9)	0.942
Coronary artery bypass grafting	2 (0.5)	1 (0.3)	0 (0)	0.509
Carotid artery intervention	2 (0)	0 (0)	0 (0)	0 .228
Intervention on the arteries of the lower extremities	4 (0.9)	3 (0.9)	1 (0.4)	0.560
Data from instrumental examinations of non-coronary arteries				
Mean intima-media thickness (mm, Me [LQ ; UQ])	0.12 [0.11; 0.12]	0.12 [0.11; 0.12]	0.12 [0.12; 0.12]	0.877
Stenosis of carotid arteries 50% or more	158 (38.3)	123 (38.0)	90 (31.7)	0.089
Stenosis of the arteries of the lower extremities	132 (32.0)	91 (28.1)	94 (33.1)	0.109

*Notes: CMD – carbohydrate metabolism disorders, DM – diabetes mellitus, CABG – coronary artery bypass grafting, * unless otherwise stated; , p 1-2, 2-3, 1-3 - p in pairwise comparison of groups 1-2, 2-3, 1-3*

Supplementary Table S2. Medical therapy for diabetes mellitus (prehospital *)

Group of drugs	(n, %)*
Oral medications for the treatment of diabetes mellitus (n, %)	216 (76.1)
Metformin (n, %)	134 (47.2)
Sulfonylurea drugs (n, %)	137 (48.2)
DPP-4 inhibitors (n, %)	11 (3.9)
SGLT-2 inhibitor (n, %)	9 (3.2)
GLP-1 agonists (n, %)	3 (1.1)
Prehospital insulin (U, Me [LQ ; UQ])	42 (14.8)
Hospital insulin (ED, Me [LQ ; UQ])	138 (48.6)

*Note: * unless otherwise stated, DPP -4 - dipeptidyl peptidase 4, SGLT2 - Sodium glucose co - transporter 2, GLP -1 - glucagon - like peptide 1.*
